# Boron bridging of rhamnogalacturonan-II, monitored by gel electrophoresis, occurs during polysaccharide synthesis and secretion but not post-secretion

**DOI:** 10.1111/tpj.12403

**Published:** 2013-12-09

**Authors:** Dimitra Chormova, David J Messenger, Stephen C Fry

**Affiliations:** The Edinburgh Cell Wall Group, Institute of Molecular Plant Sciences, School of Biological Sciences, The University of EdinburghThe King's Buildings, Mayfield Road, Edinburgh, EH9 3JH, UK

**Keywords:** rhamnogalacturonan-II, gel electrophoresis, pectin, boron, radiolabelling, cross-linking, apoplast, cell wall, *Rosa* sp., *Arabidopsis thaliana*

## Abstract

The cell-wall pectic domain rhamnogalacturonan-II (RG-II) is cross-linked via borate diester bridges, which influence the expansion, thickness and porosity of the wall. Previously, little was known about the mechanism or subcellular site of this cross-linking. Using polyacrylamide gel electrophoresis (PAGE) to separate monomeric from dimeric (boron-bridged) RG-II, we confirmed that Pb^2+^ promotes H_3_BO_3_-dependent dimerisation *in vitro*. H_3_BO_3_ concentrations as high as 50 mm did not prevent cross-linking. For *in-vivo* experiments, we successfully cultured ‘Paul's Scarlet’ rose (*Rosa* sp.) cells in boron-free medium: their wall-bound pectin contained monomeric RG-II domains but no detectable dimers. Thus pectins containing RG-II domains can be held in the wall other than via boron bridges. Re-addition of H_3_BO_3_ to 3.3 μm triggered a gradual appearance of RG-II dimer over 24 h but without detectable loss of existing monomers, suggesting that only newly synthesised RG-II was amenable to boron bridging. In agreement with this, *Rosa* cultures whose polysaccharide biosynthetic machinery had been compromised (by carbon starvation, respiratory inhibitors, anaerobiosis, freezing or boiling) lost the ability to generate RG-II dimers. We conclude that RG-II normally becomes boron-bridged during synthesis or secretion but not post-secretion. Supporting this conclusion, exogenous [^3^H]RG-II was neither dimerised in the medium nor cross-linked to existing wall-associated RG-II domains when added to *Rosa* cultures. In conclusion, in cultured *Rosa* cells RG-II domains have a brief window of opportunity for boron-bridging intraprotoplasmically or during secretion, but secretion into the apoplast is a point of no return beyond which additional boron-bridging does not readily occur.

## Introduction

Unlike most organisms, plants have a readily demonstrable requirement for boron (B) (Blevins and Lukaszewski, 1998; Goldbach and Wimmer, 2007). Boron in soil is available to plants as soluble boric acid, H_3_BO_3_, a weak Lewis acid which forms the borate anion [B(OH)_4_^−^] only at high pH (p*K*_a_ 9.1). Symptoms of B deficiency include short, thick stems and roots, dying growing points and rough or corky epidermal surfaces (Warington, 1923; Lehto *et al*., 2010; Wimmer and Eichert, 2013). This agriculturally important feature of plant life is poorly understood biochemically. Despite being an essential element, excess B is detrimental to plants, and there is a narrow window between concentrations giving deficiency and those (e.g. 5 mm) producing toxicity (Aquea *et al*., 2012). Problems of B deficiency can be solved with fertilisers, but excess B is an intractable agricultural problem, especially in some arid areas (Al-Mustafa *et al*., 1993). Understanding why plants require B, and the basis of its toxicity, will facilitate progress in agriculture.

Boron strongly affects the mechanical properties of plants: tissues with inadequate and excess B often feel ‘brittle’ and ‘rubbery’, respectively (Loomis and Durst, 1992; Blevins and Lukaszewski, 1998). This mechanical effect suggests a role for B in cell-wall structure, as does the observation that withdrawal of B decreases the elasticity of root cell walls within 5 min (Findeklee and Goldbach, 1996). Pectin-rich tissues (e.g. collenchyma) show especially striking deficiency symptoms, and the B requirements of different plants correlate with their pectin contents (Hu *et al*., 1996). Loomis and Durst (1992) first suggested that apiose (Api*) was the key wall component to which B binds, and it is now widely accepted that pectic Api residues are a plant-specific, B-dependent wall component.

Pectins are partially methylesterified, α-GalA-rich cell-wall polysaccharides. They are built of up to four domains [homogalacturonan (HGA), rhamnogalacturonans (RG-I, RG-II) and xylogalacturonan], which are glycosidically interlinked (Ishii *et al*., 2001; Coenen *et al*., 2007). When, for analytical purposes, pectin is de-esterified and then digested with endopolygalacturonase (EPG), the HGA domain is degraded to free GalA plus di- and tri-galacturonide, whereas RG-I and RG-II are released intact and can be purified by gel-permeation chromatography (Matoh *et al*., 1993, 1996; Coenen *et al*., 2007).

Rhamnogalacturonan-II is of particular interest because of its ability to form borate esters. Rhamnogalacturonan-II is a small [usual degree of polymerisation (DP) 29–30; about 5 kDa] but complex, taxonomically conserved, pectic domain that is ubiquitous in the primary cell walls of vascular plants. To its acidic backbone of about eight α-GalA residues are attached five unique sidechains (O'Neill *et al*., 2004; Pabst *et al*., 2013): (i) **A** (octasaccharide) composed of α-l-Gal, β-GlcA (sometimes methylesterified), α-MeXyl, α-Fuc, β-Rha, α-GalA, β-GalA (carrying zero to two methyl ether groups), β-Api; (ii) **B** (hexa- to nonasaccharide) of β-Ara*f* (zero to one), α-Rha (zero to two), α-Ara*p*, β-d-Gal, α-MeFuc acetyl ester, α-AceA acetyl ester, β-Rha, β-Api; (iii) **C** (disaccharide) of α-Rha, α-Kdo; (iv) **D** (disaccharide) of β-Ara*f*, β-Dha; (v) and ‘**E**’ (monomer), α-Ara*f*. The RG-II domain carries *O*-acetyl esters in sidechain **B** (O'Neill *et al*., 2004), but only the single GlcA residue of sidechain **A** is methylesterified (Pabst *et al*., 2013), so sidechains **A**–**D** are negatively charged. Sidechain **A** has a unique ability to strongly bond to H_3_BO_3_.

Driselase digestion of plant cell walls yields a stable B–RG-II complex (Matoh *et al*., 1993, 1996; Kobayashi *et al*., 1996). In B-sufficient tissues, many of the wall's RG-II domains are dimerised by tetrahedral B-bridges involving the O-2 + O-3 of two sidechain-**A** Api residues in a diol–(B^−^)–diol diester arrangement (Kobayashi *et al*., 1996; O'Neill *et al*., 1996, 2004; Ishii *et al*., 2002). Such dimers are scarce in the *bor1* mutant (defective in H_3_BO_3_ transport) (Noguchi *et al*., 2003) and in B-starved wild-type plants. The need to form a *precise* B-bridge may be why the structure of RG-II is highly conserved. For example, B-bridging of RG-II is diminished in the tobacco mutant *nolac-H18*, which is defective in NpGUT1 (glucuronosyltransferase) and consequently lacks GlcA and l-Gal in sidechain **A** (Iwai *et al*., 2002) (notwithstanding the curious fact that two Arabidopsis proteins, IRX10 and IRX10-L, which resemble N-terminal truncated versions of NpGUT1, appear to contribute in Arabidopsis to the biosynthesis of xylan backbones rather than RG-II; Wu *et al*., 2009). Furthermore, *mur1* [which has l-Gal in place of l-Fuc (Reuhs *et al*., 2004) and may have a shortened sidechain **A** (Pabst *et al*., 2013)] is defective in RG-II B-bridging (O'Neill *et al*., 2001). Finally, virus-induced gene silencing (VIGS) of *AXS1* (leading to Api deficiency) also compromises RG-II bridging (Ahn *et al*., 2006). The formation of RG-II–(B^−^)–RG-II bridges is a major reason why plants require B, and why the pectin-poor Poales need less B than dicots.

Functionally, RG-II bridging via B decreases the size of the pores in the wall (Fleischer *et al*., 1998, 1999), affecting intercellular communication. It also affects the mechanical properties and thickness pf the wall and the plant's growth and morphogenesis (Hirsch and Torrey, 1980; Hu and Brown, 1994; Findeklee and Goldbach, 1996; Ishii *et al*., 2001). For example, pollen genetically unable to make Kdo (unique to RG-II) is defective in pollen-tube growth (Delmas *et al*., 2008) and *AXS1*-silenced plants and the mutants *bor1* and *mur1* are dwarfed, suggesting that B-bridging is necessary for normal growth and morphogenesis. However, with our current understanding of B-bridges largely limited to a static description of their chemistry, it is unclear why increasing the cross-linking of a wall component would *favour* cell expansion, which is dependent on wall loosening. The kinetics of B-bridge formation and turnover await elucidation.

Most neutral sugars rapidly esterify with the borate anion at a pH of about 9, a fact exploited in the electrophoresis of ‘neutral’ sugars (Weigel, 1963; Goubet *et al*., 2006), but the bonds formed are unstable at pH < 7, characteristic of the cell wall. Such bonds are thus *not* valid models of B–RG-II bridging. Furanosyl *cis*-1,2-diols (e.g. Rib*f* in NAD^+^, Api*f* in methyl β-apioside, and hydrated 1-deoxy-3-keto-l-ribulose, Chen *et al*., 2002) form B esters that are more stable than their *trans*-diol or pyranosyl counterparts (Ishii and Ono, 1999), but even these are unstable compared with B–RG-II bridges. The latter are stable enough to withstand column chromatography in (or dialysis against) mildly acidic, B-free buffers (half-life ≈ 24 h at pH 2.8 and 20°C) (O'Neill *et al*., 1996).

While slow to *break*, B–RG-II bridges are also slow to *form in vitro* with pure RG-II + H_3_BO_3_ as substrates. Such bridging is slightly promoted by very high Ca^2+^, e.g. 50 mm (Ishii *et al*., 1999). Also, some non-biological cations (e.g. 0.5 mm Pb^2+^, Sr^2+^ or Ba^2+^) strongly enhance RG-II bridging by H_3_BO_3_ *in vitro* (O'Neill *et al*., 1996; Ishii *et al*., 1999); it remains unknown what biological agent ‘replaces’ Pb^2+^ etc. *in vivo*.

It was reported that when H_3_BO_3_ is resupplied to B-starved *Chenopodium* cells (Fleischer *et al*., 1999) or *Cucurbita* leaves (Ishii *et al*., 2001), many of the existing RG-II domains rapidly became B-bridged. This suggests that B-bridging *can* occur in the wall long after pectin secretion. However, it was not known if this is the normal subcellular site of bridge formation in B-sufficient cells – alternatives being within the Golgi system prior to (or at the plasma membrane during) pectin secretion. Resolving this question would inform our attempts to detect enzymes and other components needed for promoting B-bridging *in vivo*.

Little is known about why excess B is toxic to plants (Loomis and Durst, 1992; Reid *et al*., 2004), but the effect of a high [B] on tissue mechanics points to an involvement of the cell wall, probably RG-II. We considered the hypothesis that the H_3_BO_3_:RG-II molar ratio is critical. At a H_3_BO_3_:RG-II ratio of zero, all the RG-II molecules in a population will clearly be monomeric (represented in the equations below as RG-II.H_2_, where the two H atoms indicated are those of the *cis*-diol of the Api residue in sidechain **A**); at a ratio of 0.5, most of the molecules can dimerise, perhaps via two steps:


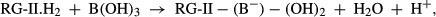


then


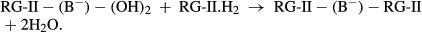


But at a ratio of 1.0 or higher, most of the RG-II molecules might quickly become ‘half-bridged’ [as RG-II–(B^−^)–(OH)_2_] and thus locked in the monomeric form:


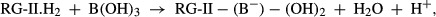


unable to find a B-free partner with which to form a full bridge:


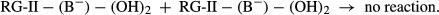


This is a potential explanation of why high H_3_BO_3_ concentrations are toxic to plants.

The main objectives of this work were to define when in the ‘career’ of an RG-II domain the B-bridging occurs *in vivo*, and whether excess B concentrations interfere in bridging. Secondarily, we introduced several methodological innovations: (i) to provide useful biological material for these studies, we developed a *Rosa* cell-suspension culture capable of growing in a B-free medium and thus producing only non-B-bridged RG-II; (ii) we prepared high-specific-activity radiolabelled RG-II; and (iii) we developed a polyacrylamide gel electrophoresis (PAGE) system for separating monomeric and dimeric RG-II. Using these techniques, we now report on the *in-vivo* B-bridging of endogenous and exogenous RG-II.

## Results

### Separation of monomeric and dimeric RG-II by gel electrophoresis

**Figure 1:**
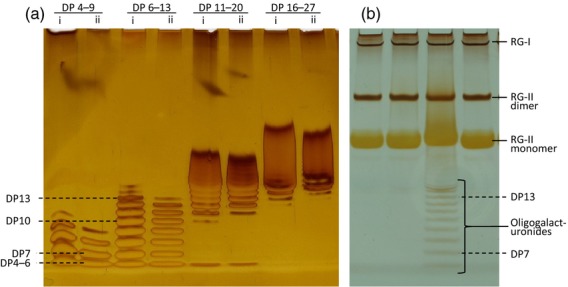
Resolution of rhamnogalacturonans and oligogalacturonides by gel electrophoresis.(a) Oligogalacturonide preparations of the approximate degree of polymerisation (DP) ranges indicated were loaded at (i) 0.50% or (ii) 0.25% w/v.(b) Endopolygalacturonase digestion products of *Rosa* cell walls. The third sample from the left shows the result of incomplete digestion of the homogalacturonan.

Previous work on RG-II cross-linking has employed anion-exchange and gel-permeation chromatography combined with inductively coupled plasma mass spectrometry (ICP–MS) to separate monomers from dimers and to quantify them (Kobayashi *et al*., 1996; O'Neill *et al*., 1996; Fleischer *et al*., 1999; Matsunaga *et al*., 2004). To allow us to run multiple samples simultaneously, we developed a PAGE system. Monomeric and dimeric RG-II have a similar charge:mass ratio, but the sieving properties of polyacrylamide enabled their separation by size (about 5 and 10 kDa respectively), as with protein SDS–PAGE and oligosaccharide polysaccharide analysis using carbohydrate gel electrophoresis (Goubet *et al*., 2006). Advantages of gel electrophoresis include excellent resolution, convenient long-term storage of separated samples and simple radioisotope detection. The developed system was able to separate RG-I, RG-II dimer, RG-II monomer and oligogalacturonides (Figure 1). The bromophenol blue marker (not visible in Figure 1 because it elutes during staining) runs slightly slower than the smallest oligogalacturonides. All oligogalacturonides of DP < 20 were well resolved from RG-II.

**Figure 2:**
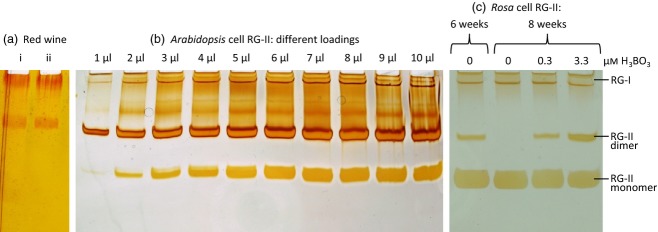
Characterisation of rhamnogalacturonan-II (RG-II) preparations by gel electrophoresis. (a) Non-volatile solutes of red wine were dissolved at 1% (w/v) in water and electrophoresed before (i) and after (ii) 24 h of dialysis. (b) Electrophoresis of various volumes of a RG-II-rich preparation from Arabidopsis cultures grown in standard medium (containing 100 μm H_3_BO_3_). The 1-μl sample contained about 0.2 μg RG-II. (c) *Rosa* cells were grown with 0, 0.3 or 3.3 μm H_3_BO_3_ for various periods, the medium being renewed fortnightly. Rhamnogalacturonan-II from their cell walls was electrophoresed.

**Figure 3:**
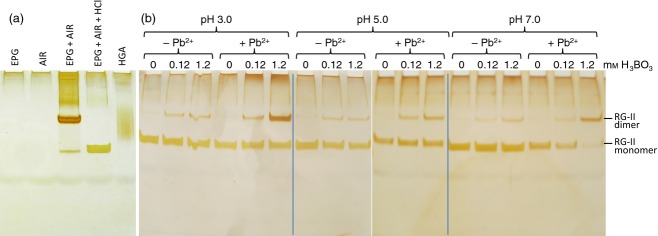
Artificially monomerising dimer and dimerising monomer.(a) Arabidopsis alcohol-insoluble residue (AIR) was saponified with Na_2_CO_3_, then incubated for 16 h with or without endopolygalacturonase (EPG); products were analysed by PAGE. Lane 4 shows the products of an additional 16-h incubation in 0.1 m HCl. Endopolygalacturonase alone gave no rhamnogalacturonan-II (RG-II). HGA, homogalacturonan (‘polygalacturonic acid’; marker). (b) Monomeric RG-II, produced as in lane 4 of (a), was incubated for 16 h in 0, 0.12 or 1.2 mm H_3_BO_3_, in the presence or absence of 0.5 mm PbNO_3_. The solutions were buffered at pH 3, 5 or 7.

Optimum staining of RG-II was achieved with a silver method similar to that used for staining proteins (Nesterenko *et al*., 1994; Chevallet *et al*., 2006; Simpson, 2007; Singh, 2011); alcian blue and basic fuchsin failed to stain rhamnogalacturonans and oligogalacturonides satisfactorily. Staining intensity was related to the amount of RG-II loaded (Figure 2b). The RG-II dimer stained more intensely than the monomer (Figure 3).

### Characterisation and radiolabelling of RG-II

Cell walls (as alcohol-insoluble residue, AIR) of cell-suspension cultures were de-methylesterified with Na_2_CO_3_, then digested with EPG, generating rhamnogalacturonans (Figures 2b and 3a) plus non-staining oligogalacturonides. Rhamnogalacturonan-II freshly prepared from red wine or from the cell walls of Arabidopsis cell cultures grown in standard medium (containing 100 μm H_3_BO_3_) was largely dimeric (Figure 2a,b), as expected (O'Neill *et al*., 2004), whereas RG-II from *Rosa* cell cultures grown in their routine medium (containing 3.3 μm H_3_BO_3_) produced RG-II that was only partially dimeric (Figure 2c, right-hand lane). Similar results were obtained when AIR was digested with Driselase or impure pectinase preparations. Driselase released arabinogalactan–protein fragments in addition to RG-II and was therefore not routinely used.

**Figure 4:**
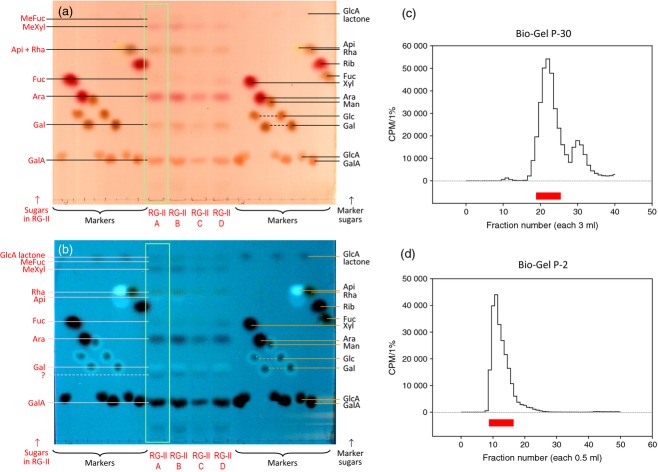
Characterisation and radiolabelling of rhamnogalacturonan-II (RG-II).(a), (b) Four independent preparations of *Rosa* RG-II, A–D, were acid hydrolysed and the products separated by TLC and stained with aniline hydrogen phthalate. The plate was photographed under visible light (a) and 360-nm ultraviolet light (b). Preparation A was then radiolabelled with NaB^3^H_4_ and the ^3^H-polysaccharide was purified by gel-permeation chromatography on Bio-Gel P-30 (c) followed by Bio-Gel P-2 (d). In each case, the fractions indicated in red were harvested.

For preparative purposes, Arabidopsis or *Rosa* AIR was de-esterified then EPG-digested, and the RG-II purified from the crude digest by gel-permeation chromatography. Four independent preparations of *Rosa* RG-II (A–D) were analysed for sugar composition (Figure 4a,b and Figure S1). In each case, prominent monosaccharides were GalA, Gal, Ara, Rha, MeXyl, Fuc and Api; smaller amounts of MeFuc and GlcA lactone (de-lactonised during the HPLC run) were also detected. This agrees with the published composition of RG-II (O'Neill *et al*., 2004). A minor sugar migrating slightly slower than Gal on TLC and several peaks on the HPLC remain unidentified. 2-Keto-3-deoxy-d-manno-octulosonic acid (Kdo) had an HPLC retention time of 74.0 min, but authentic Kdo was completely degraded during acid hydrolysis, as reported by York *et al*. (1985).

Preparation ‘A’ was selected for radiolabelling with NaB^3^H_4_. The crude [^3^H]RG-II was repurified by gel-permeation chromatography (Figure 4c,d). On PAGE, the purified product, after monomerisation with HCl, gave a single band detectable by fluorography.

### Artificially monomerising dimer and dimerising monomer

In agreement with previous reports (O'Neill *et al*., 1996; Matsunaga *et al*., 2004; Yapo, 2011), and validating our electrophoresis method, we showed that dimeric RG-II was monomerised at pH 1 (Figure 3a). Under the conditions used, there was no evidence of degradation to smaller products such as might occur if the HCl cleaved the highly labile apiosyl linkages. The loadings in lanes 3 and 4 of Figure 3(a) are equal, yet the staining intensity of monomer is noticeably weaker than that of the starting dimer, confirming that the silver stain is more sensitive to the dimer.

We also showed that monomeric RG-II can be dimerised by treatment with 0.1–1.0 mm H_3_BO_3_ (Figure 3b). Boron-dependent dimerisation was little affected by pH in the range 3–7, but was strongly promoted by 0.5 mm Pb^2+^, as reported before (O'Neill *et al*., 1996).

### Acclimation of *Rosa* cells to B-free medium

To provide plant cells suitable for monitoring the *in-vivo* cross-linking of monomeric RG-II, we attempted to grow *Rosa*, Arabidopsis and *Spinacia* cell suspension cultures in their respective media adjusted to contain ‘0’, 10 or 100% of the standard H_3_BO_3_ concentration. Polypropylene flasks were used, avoiding contamination from B in glassware. After 3–4 days, Arabidopsis and *Spinacia* cells stopped growing and died in media containing ‘0’ or 10 μm H_3_BO_3_. The *Rosa* culture, in contrast, continued to grow well at ‘0’, 0.33 and 3.3 μm H_3_BO_3_, the only noticeable difference being that within 2–4 weeks the ‘zero-B’ cells became whitish instead of pale yellow. The medium was renewed fortnightly: after 6 weeks in ‘zero-B’ medium, the *Rosa* cells still contained appreciable RG-II dimer, but this became undetectable by 8 weeks (Figure 2c).The walls of B-free cells still contained approximately normal amounts of RG-II, albeit all monomeric. The pectins containing these RG-II domains were firmly linked in the cell wall, unlike those in B-free *Chenopodium* cells, which dissolved in phosphate buffer (Fleischer *et al*., 1999). The *Rosa* cells have now been successfully maintained in our laboratory in the absence of deliberately added B for over 2 years.

Only traces of contaminating B were present in our media. The ‘0’, 10 and 100% [B] *Rosa* media were shown by ICP–MS to contain 0.95, 2.71 and 29.5 μg L^−1^ total B; theoretical values are 0, 3.5 and 35 μg L^−1^. Thus, the ‘zero-B’ medium contained about 88 nm B, presumably as a contaminant from the other nutrients, but this amount was too low for detectable formation of RG-II dimers.

### Endogenous RG-II domains have only a brief window of opportunity for dimerisation in *Rosa* cell cultures

**Figure 5:**
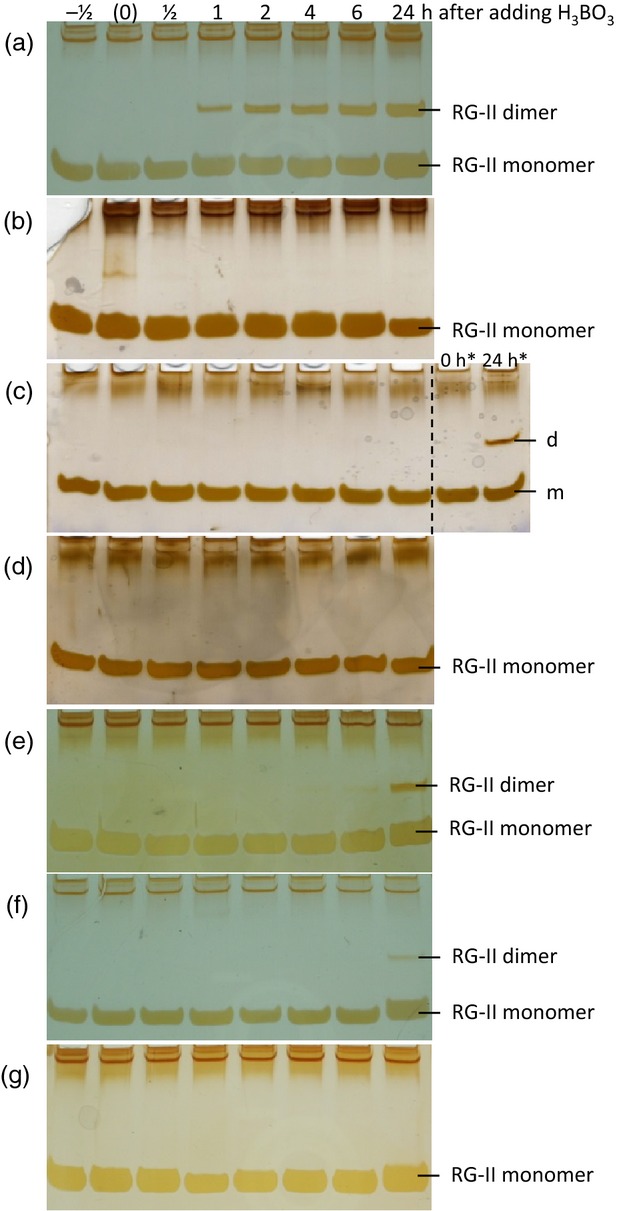
Production of rhamnogalacturonan-II (RG-II) dimer in *Rosa* cells is dependent on *de-novo* polysaccharide biosynthesis.*Rosa* cells maintained in B-free medium for several months were re-fed H_3_BO_3_ to 3.3 μm at time ‘0’. The cells were (a) healthy, (b) pre-starved of their usual carbon source for 4 days, (c) treated with 10 μm carbonyl cyanide 3-chlorophenylhydrazone (or with an equivalent volume of ethanol, indicated by *), (d) treated with 200 μm 2,4-dinitrophenol, (e) anaerobic, (f) frozen/thawed, or (g) boiled. In each case, samples of alcohol-insoluble residue taken at intervals after H_3_BO_3_ addition were saponified and digested by endopolygalacturonase, and products were analysed by PAGE. Time ‘–½’ represents a sample taken 30 min before the addition of H_3_BO_3_; time ‘(0)’ was sampled as quickly as possible after H_3_BO_3_ addition.

To trace the possible dimerisation of wall-bound monomeric RG-II domains *in vivo*, we re-fed 3.3 μm H_3_BO_3_ to zero-B *Rosa* cell cultures that contained no detectable RG-II dimers. No dimer appeared during the first 30 min of H_3_BO_3_ addition (Figure 5a), in contrast to the report on *Chenopodium* cells (Fleischer *et al*., 1999). Over the following 24 h, RG-II dimers did gradually form, but with no concurrent loss of monomer; indeed, by 24 h there had been a noticeable increase in total RG-II (Figure 5a). This suggests that previously wall-bound monomeric RG-II domains were unable subsequently to dimerise in the presence of 3.3 μm H_3_BO_3_, but that RG-II newly synthesised since the addition of H_3_BO_3_ was able to dimerise.

To test this interpretation, we applied various treatments designed to decrease or prevent *de-novo* polysaccharide synthesis and then resupplied 3.3 μm H_3_BO_3_. Each such treatment strongly diminished the production of dimeric RG-II (Figure 5b–f). Cells starved of glycerol, their usual carbon source, for 4 days (and thus unable to produce new polysaccharides) and cells treated with the respiratory inhibitors carbonyl cyanide 3-chlorophenylhydrazone (CCCP) and 2,4-dinitrophenol (DNP) produced no detectable dimer (Figure 5b–d), although controls did generate some dimer within 24 h (Figure 5c). Living *Rosa* cells incubated with reduced aeration produced little dimer within 24 h, frozen–thawed cells produced very little and boiled cells produced none (Figure 5e–g).

Thus, *Rosa* cells re-fed 3.3 μm H_3_BO_3_ were only able to dimerise RG-II efficiently when concurrent production and secretion of polysaccharide was occurring. We conclude that B-bridging of RG-II normally occurs during or very shortly after *de novo* biosynthesis, and that secretion into the wall is a point of no return precluding subsequent dimerisation.

### Exogenous RG-II is not dimerised in cell walls or culture medium of *Rosa* cells

**Figure 6:**
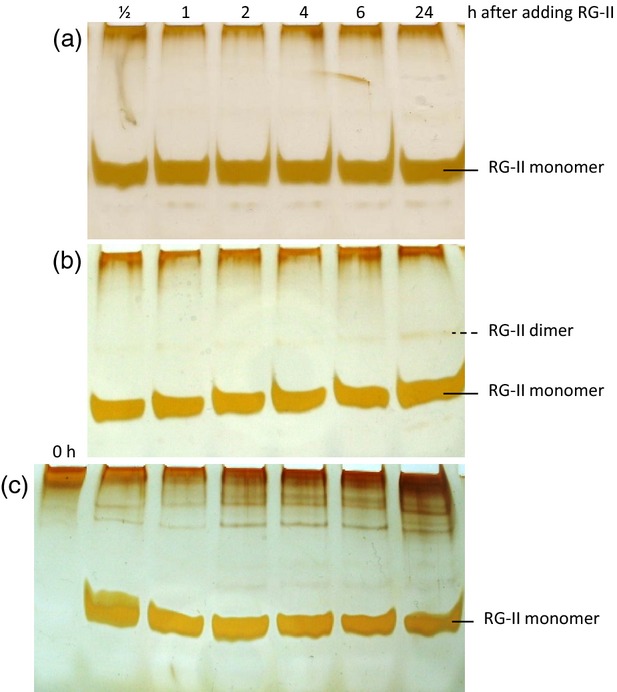
Rhamnogalacturonan-II (RG-II) largely fails to dimerise in *Rosa* culture apoplast. Purified monomeric RG-II (about 60 μm) was incubated in cell-free spent medium harvested from a zero-B *Rosa* culture 4 days after subculture. (a) No H_3_BO_3_, (b) H_3_BO_3_ added to 1.2 mm concurrently with the RG-II, (c) cells and 1.2 mm H_3_BO_3_ added. At intervals (0–24 h), samples of the medium were frozen and later electrophoresed.

Supporting the conclusion that the dimerisation of endogenous RG-II domains normally occurs intraprotoplasmically and/or during secretion, we found that exogenous monomeric RG-II (approximately 60 μm) remained soluble, and monomeric, in spent culture medium. Only a trace of dimer was observed when 1.2 mm H_3_BO_3_ was added to the medium (Figure 6b) compared with a zero-B sample (Figure 6a). Thus, there was no evidence for the presence of secreted factors, such as enzymes, B carriers or RG-II chaperones, that might ‘mimic’ Pb^2+^ to enhance apoplastic RG-II dimerisation *in vivo*. In addition, the presence of live cells in the medium had no effect on the behaviour of soluble extracellular RG-II (Figure 6c), indicating the absence of wall-bound factors that might act as immobilised catalysts promoting the dimerisation of soluble RG-II.

**Figure 7:**
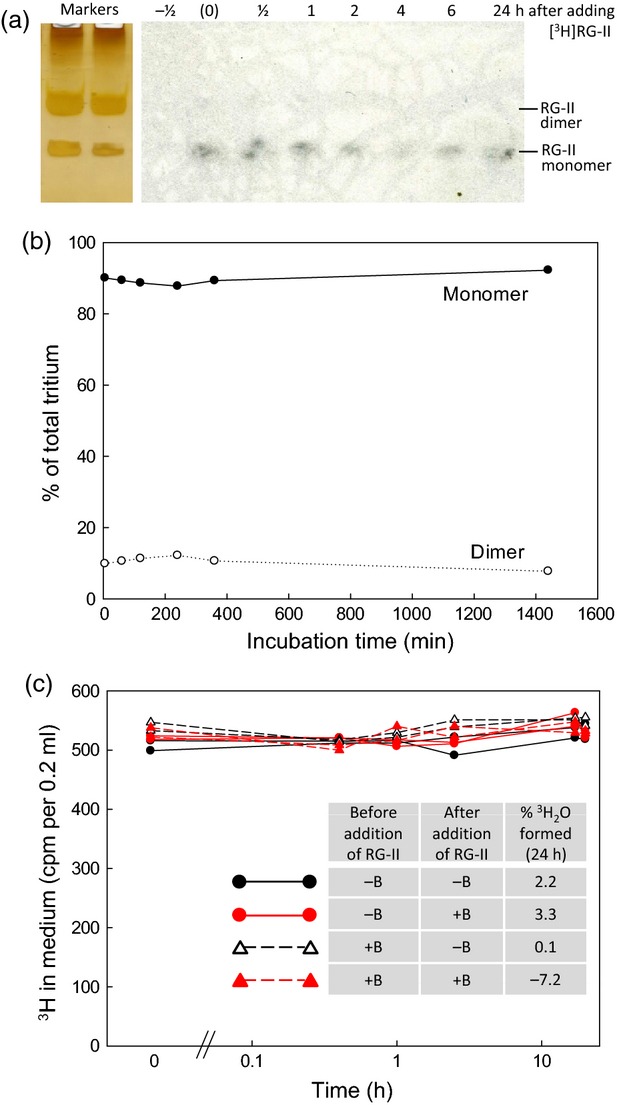
Exogenous [^3^H] rhamnogalacturonan-II (RG-II) fails to dimerise or integrate into walls in cultured *Rosa* cells. (a), (b) Monomeric [^3^H]RG-II (3.9 μm) was fed to B-starved 4-day-old *Rosa* cultures at the same time as 1.2 mm H_3_BO_3_. Samples of medium were electrophoresed: (a) fluorograph; (b) relevant bands scintillation counted. (c) In a separate experiment, 8.5 nm [^3^H]RG-II was fed to B-sufficient and B-deficient *Rosa* cultures, with or without 3.3 μm H_3_BO_3_ supplementation. At intervals, samples of medium were assayed for total remaining soluble ^3^H (graph); at 24 h, additional samples were assayed for volatile ^3^H (inset Table; indicating any ^3^H_2_O formed by catabolism).

It might be objected that the concentration of RG-II (about 60 μm) used in Figure 6 had saturated any biological B-bridging mechanism (enzymes, B transfer agents etc.) so that only a small percentage of the added RG-II was successfully dimerised. We therefore also tested a tracer concentration (3.9 μm) of monomeric radiolabelled RG-II (Figure 7). No dimerisation of exogenous soluble [^3^H]RG-II was observed in the presence of B-supplemented *Rosa* cultures (Figure 7a,b). Similar results were obtained when the [^3^H]RG-II was mixed with cell-free spent medium harvested from similar cells (Figure S2). Furthermore, all the [^3^H]RG-II remained soluble in the medium (Figure 7c); the cells, collected after 24 h in the presence of [^3^H]RG-II and thoroughly washed in water, showed no bound radioactivity. This result was obtained with all four permutations of cells pre-grown with or without H_3_BO_3_ and then fed [^3^H]RG-II with or without H_3_BO_3_ (Figure 7c). Samples of medium collected at 24 h contained negligible ^3^H_2_O (Fig. 7c inset), confirming that the [^3^H]galactonate moiety of the [^3^H]RG-II was not being catabolised.

It could be argued that bridging of soluble extracellular RG-II to cell walls was inefficient owing to its dilution into a relatively large volume of medium; however, a similar lack of bridging was observed when a very small volume of radioactive RG-II solution was pipetted directly on to a small mound of *Rosa* cells growing on agar with no free liquid medium. Therefore, dilution into the medium was not the cause of the failure of wall binding.

Thus, cultured *Rosa* cells were unable to ‘mimic’ Pb^2+^ by catalysing the dimerisation of extracellular RG-II in the presence of H_3_BO_3_; and B-starved *Rosa* cells were unable to link exogenous free RG-II to their own wall-associated monomeric RG-II domains, even with the benefit of B supplementation.

### Excess boric acid does not prevent RG-II dimerisation – eliminating a potential basis of B toxicity

It might be speculated that since the concentration of H_3_BO_3_ used in some experiments (Figures 6 and 7) was high (1.2 mm), greatly exceeding that of RG-II, dimerisation might have been inhibited, as proposed in the Introduction. However, in an *in-vitro* cross-linking experiment similar to that in Figure 3(b), dimer production was not inhibited by H_3_BO_3_ concentrations up to at least 50 mm (Table 1). Thus H_3_BO_3_ at the highest concentration used in our *in-vivo* experiments (1.2 mm) did not interfere in RG-II cross-linking.

**Table 1:** Effect of high H_3_BO_3_ concentrations on the Pb^2+^-induced dimerisation of [^3^H] rhamnogalacturonan-II (RG-II). Partially monomerised [^3^H]RG-II (22 μm) was incubated for 16 h in 0.2 m succinate buffer (Na^+^), pH 5.5, in the presence of 0.5 mm Pb(NO_3_)_2_ plus the indicated concentration of boric acid, and then analysed by PAGE for radioactive dimers

Added H_3_BO_3_ concentration (mm)	Increase in yield of dimeric [^3^H]RG-II (as % of total tritium)
0	0.0^a^
0.2	1.0
0.4	1.9
0.8	5.6
1.6	9.3
3.1	10.6
6.3	12.7
12.5	16.8
25.0	18.5
50.0	19.7

a In this experiment, monomerisation of the RG-II was incomplete; in the H_3_BO_3_-untreated control, 44.8% of the total ^3^H was in the form of monomeric RG-II.

## Discussion

### Boron bridges are not essential for holding RG-II domains in the wall

It is widely accepted that RG-II is a cell-wall pectin domain, glycosidically linked between other pectic domains (especially HGA and RG-I) by α-(1→4)-galacturonosyl bonds. Sidechain **A** endows RG-II with the ability to form unusually durable tetrahedral B-bridges at typical apoplastic pH values (e.g. 4–5), and such bridging has been shown to be essential for the biophysical properties of the cell wall, and thus for cell growth and development. Although the B-bridges may help to hold the pectin within the wall architecture, our data show that their existence is not essential for this: *Rosa* cells grown in the absence of B still produce pectin that contains monomeric RG-II domains, and this pectin remains as an integral component of the cell wall upon washing in Na_2_CO_3_. It is likely that cross-links involving other pectic domains ensure this wall association, e.g. Ca^2+^ bridges between acidic HGA domains, glycosidic bonds between xyloglucan and RG-I (Popper and Fry, 2005) and possibly galacturonoyl esters or amides to other wall components (Kim and Carpita, 1992; Brown and Fry, 1993; Perrone *et al*., 1998). Nevertheless, the absence of B, or a mutation rendering the RG-II incapable of binding B, results in cell walls with defective biophysical properties (Fleischer *et al*., 1998; O'Neill *et al*., 2001; Noguchi *et al*., 2003).

### Dimerisation of RG-II is largely protoplasmic, not apoplastic

Although it is clear that B-bridges exist between RG-II domains, and that their existence is biologically important, very little was known about the mechanism of bridge formation, or at what stage(s) during the ‘career’ of a RG-II domain it is amenable to being dimerised *in vivo*. Possibilities include synthesis in the Golgi bodies during *de-novo* biosynthesis, during trafficking to the plasma membrane, upon exocytosis into the wall and during maturation within the wall. Dimerisation of RG-II is a slow process *in vitro* unless non-biological agents such as Pb^2+^, Sr^2+^ or very high Ca^2+^ are added. Yet dimerisation appears to occur efficiently *in-vivo*, even in the presence of low H_3_BO_3_ concentrations such as the 3.3 μm which is routinely present in *Rosa* medium. There are important differences between *in-vivo* and *in-vitro* dimerisation, e.g. all *in-vitro* experiments to date have used purified free RG-II (Kobayashi *et al*., 1996; O'Neill *et al*., 1996), whereas *in vivo* the RG-II occurs as domains within much larger pectin molecules. Also, RG-II preparations used for B-bridging experiments *in vitro* have all used de-esterified RG-II (O'Neill *et al*., 1996), which will lack the methylester group of on the GlcA residue of sidechain **A** and the *O*-acetyl ester groups of sidechain **B**. Furthermore, *in-vitro* experiments have been conducted in the absence of enzymes and of any cellular components that might act as carriers of B, RG-II chaperones or catalysts of the borate esterification reaction. For all these reasons, it is highly informative to monitor RG-II dimerisation *in-vivo* in comparison with *in-vitro* dimerisation. In the present paper, we have studied the dimerisation of both endogenous RG-II domains (covalently linked to other pectic domains, retaining the acetyl esters and associated with all other normal cellular components) and exogenous RG-II (assured to be extracellular, of a known concentration and if desired radioactively labelled for ease of quantification).

These distinct approaches tracking endogenous pectin-bound RG-II domains and exogenous free RG-II led to the same conclusion: RG-II is not readily dimerised in the apoplast, and protoplasmic dimerisation is dependent on concurrent synthesis and/or secretion of polysaccharides. Thus, when H_3_BO_3_ was re-added to a B-free culture, endogenous RG-II dimer appeared only slowly, over a period of 24 h, at a rate commensurate with *de-novo* synthesis. There was no disappearance of the large existing pool of wall-bound monomeric RG-II domains. Furthermore, prevention of polysaccharide biosynthesis blocked the accumulation of RG-II dimer, supporting the conclusion that B-bridging of RG-II occurs intraprotoplasmically and/or at the time of secretion, but not appreciably later. It is possible that the B-bridging occurs within the Golgi cisternae or within the Golgi-derived vesicles *en route* to the plasma membrane; it is also possible that B-bridging occurs at the moment of exocytosis, when the RG-II first comes into contact with the plasma membrane. We conclude that at the time of integration into the cell wall, many pectin molecules are already B-bridged via their RG-II domains. Golgi and exocytotic sites of dimerisation would not be accessible to exogenous RG-II, added to the culture medium, in accordance with the inability of the cells to cross-link soluble extracellular free RG-II or to bind it to their existing wall-bound RG-II domains.

It had been reported that when 10–100 μm H_3_BO_3_ is resupplied to B-starved *Chenopodium* cells (Fleischer *et al*., 1999), many of the existing RG-II domains rapidly (<10 min) become B-bridged. Our observations do not agree with this. The reason for the discrepancy is unclear; however, it is surprising that in the *Chenopodium* cells 90% of the endogenous high-M_r_ pectin-associated RG-II domains were extractable in cold phosphate buffer (Fleischer *et al*., 1999), suggesting that they were not truly integrated within the cell wall. In contrast, we found that B-free *Rosa* cells, in which the RG-II-domain-containing pectins were firmly bound within the wall, showed no dimer production during the first 30 min of restoring the cells' usual H_3_BO_3_ concentration. Another difference between the *Rosa* and *Chenopodium* cells was that the latter required subculturing every 2 days so that they did not enter the stationary phase. It was reported that if they did enter the stationary phase the *Chenopodium* cells failed to stop expanding and eventually burst (Fleischer *et al*., 1999). Our B-free *Rosa* cells in contrast were routinely subcultured every 2 weeks, and survived for at least 3 weeks if they were not subcultured. They may have become better acclimated to a B-free environment thanks to having been maintained in a low [B] medium for many years (Nash and Davies, 1972).

Ishii *et al*. (2001) also reported the *in muro* dimerisation of RG-II. When 25 μm H_3_BO_3_ was supplied to B-deprived *Cucurbita* plants, the proportion of B-bridged RG-II domains in the third leaf gradually increased from 10–33% to 80–93% over a 22-h period (there is some uncertainty about the figures, depending on whether the% dimer values in Table 1 and Figure 2(b) are reported on a w/w or mol/mol basis). Ishii *et al*. (2001) suggested that pre-formed, presumably wall-localised, RG-II domains dimerised *in muro* after H_3_BO_3_ addition. However, it was not reported how much new wall biosynthesis occurred during the 22-h period of observation. It seems plausible that much of the dimeric RG-II detectable in *Cucurbita* leaves at 22 h had been biosynthesised *de novo* after H_3_BO_3_ treatment, and thus that most dimer formation may have involved newly synthesised RG-II domains and taken place intraprotoplasmically or during secretion, as indicated by our own work.

### Methodological advances

We developed an effective PAGE system for resolving monomeric and dimeric RG-II, with several advantages over existing methods such as gel-permeation and anion-exchange chromatography. Multiple samples with little or no pre-purification can be run simultaneously; resolution is excellent and rapid; detection is highly sensitive by staining; completed gels are amenable to long-term storage; detection and quantification of radioactive RG-II is facilitated; and no sophisticated apparatus is required. Furthermore the quantitative trifluoroacetic acid (TFA)/scintillation-counting method developed for assay of [^3^H]RG-II is not compromised by chemiluminescence, a common problem with radioactive bands on polyacrylamide gels (see https://www.nationaldiagnostics.com/liquid-scintillation/article/chemiluminescence-and-static-electricity, 2012).

We also developed a method for radiolabelling RG-II based on reductive tritiation with NaB^3^H_4_. This method converts the oxo- group of the reducing terminus (d-galacturonic acid in the case of RG-II) to the corresponding alcohol (in this case l-galactonic acid), in which one of the carbon-bonded H atoms is stably replaced by tritium. The radiolabelled substrate therefore has a minor chemical difference from free RG-II, but this difference concerns only one out of the 30 sugar residues of RG-II, and clearly does not compromise the ability of the RG-II to undergo H_3_BO_3_-dependent dimerisation in the presence of Pb^2+^.

### The basis of B toxicity

In the Introduction, we offered a potential explanation for the phytotoxicity of high [B]: namely that high H_3_BO_3_ favours the rapid binding by each RG-II domain of a single B atom [forming RG-II–(B^−^)–(OH)_2_], thus leaving very few B-free RG-II domains as potential partners for dimerisation. However, we found that RG-II cross-linking was not compromised *in vitro* by an approximately 2000-fold molar excess of H_3_BO_3_ (tested at up to 50 mm), so this hypothesis for the toxicity of high [B] was not supported by *in-vitro* experiments.

## Conclusion

This work shows that RG-II is not readily dimerised in the *Rosa* cell-culture apoplast, and that dimerisation is dependent on concurrent synthesis and/or secretion of polysaccharides. Thus, in these cells, RG-II domains have a brief window of opportunity for B-bridging within Golgi vesicles or during exocytosis, but secretion into the apoplast is a point of no return beyond which B-bridging does not readily occur. Further studies aimed at identifying any enzymes, B carriers or RG-II chaperones that promote B-bridging *in vivo* should therefore be focused on the endomembrane system and the plasma membrane at the site of exocytosis.

## Experimental procedures

### Gel electrophoresis

To prepare one 26.4% polyacrylamide gel of size 83 × 73 × 0.75 mm we mixed 834 μl water, 834 μl 2-amino-2-(hydroxymethyl)-1,3-propanediol (TRIS) buffer (1.5 m TRIS base, pH adjusted to 8.8 with HCl), 3.33 ml 40% (w/v) acrylamide/bis-acrylamide (29:1), 3.9 μl tetramethylethylenediamine (TEMED) and 46.7 μl of freshly prepared 0.44 m ammonium persulphate. The mixture was quickly poured and a 10-tooth comb was inserted; gelation took 30 min. The electrode buffer was 50 mm TRIS base, 38 mm glycine, pH 8.5. Samples (8 μl) were mixed with 2 μl sample buffer (0.63 m TRIS-HCl containing 0.25% (w/v) bromophenol blue and 50% (v/v) glycerol, pH 8.8).

A double-sided electrophoresis apparatus (Bio-Rad, http://www.bio-rad.com/) was used, allowing 20 samples to be run simultaneously. Electrophoresis was conducted at 200 V for 75 min. The gel was then fixed in ethanol/acetic acid/water (4:1:5) for 30 min, washed with water for 1 min three times, then treated successively with 400 μm sodium thiosulphate for exactly 1 min, water (3 × 20 sec), freshly prepared 6 mm silver nitrate in 10 μm formaldehyde for 20 min, water (2 × 20 sec) and 0.28 m Na_2_CO_3_ containing 8 μm sodium thiosulphate and 64 mm formaldehyde for 2–10 min. Colour development was stopped 30 sec before the desired intensity was reached by adding stopping solution (0.33 m TRIS base in 2% v/v acetic acid) for 10 min. Colour development continued for about 30–60 sec in the stopping solution, the background turning dark yellow (e.g. Figure 1a). Stained gels were stored in water for up to a month, or dried onto cellulose acetate sheets.

### Plant cell cultures

*Arabidopsis thaliana* (Landsberg *erecta*) suspension cultures, initiated by May and Leaver (1993), were grown under constant illumination (25 μmol m^−2^ sec^−1^) with orbital shaking at 135 r.p.m. and 25°C. Cultures were maintained at 220 ml per 500-ml flask and subcultured weekly. The medium (containing 100 μm H_3_BO_3_) was modified from May and Leaver (1993) with 2% glycerol instead of 3% sucrose.

Cell suspension cultures of ‘Paul's Scarlet’ rose (a complex hybrid; genus *Rosa*), initiated by Nickell and Tulecke (1959), were grown under constant illumination (about 10 μmol m^−2^ sec^−1^) in medium MX_1_ of Nash and Davies (1972) (containing 3.3 μm H_3_BO_3_; but with 2% glycerol instead of 2% sucrose), on an orbital shaker at 25°C as described by Fry and Street (1980).

Spinach (*Spinacia oleracea* L., cv. ‘Monstrous Viroflay’) suspension cultures, initiated by Dalton and Street (1976), were maintained in Murashige and Skoog (1962) medium (containing 100 μm H_3_BO_3_ and 1% w/v glucose) under constant illumination (60 μmol m^−2^ sec^−1^).

For a study of the tolerance of cell cultures to low B, the media were prepared from ‘AnalaR’ purity components in autoclavable polypropylene flasks (Nalgene, Thermo Scientific, http://www.thermoscientific.com/en/about-us/general-landing-page/nalgene-labware.html). H_3_BO_3_ was added at 10 or 100% of the standard concentration, or omitted altogether.

Representative samples of culture media were concentrated 10-fold, filtered, then assayed for total dissolved B by ICP–MS (we thank Dr L. J. Eades and Dr J. G. Farmer, Department of Chemistry, University of Edinburgh, UK, for conducting this analysis).

### Isolation of RG-II from cell-cultures and red wine

Cultured cells were rinsed in water, then AIR was prepared by stirring in 75% ethanol at 20°C for 4–6 h twice. The AIR was treated with 1 m Na_2_CO_3_ at 4°C for 16 h, then rinsed with water until neutral and freeze-dried. Endopolygalacturonase (10 U ml^−1^; Megazyme, http://www.megazyme.com/) was added (about 50 μl mg^−1^ AIR) and incubated at 20°C for 16 h. Solubilised material was taken for electrophoresis. In preliminary experiments, crude pectinase preparations (Sigma-Aldrich, http://www.sigma-aldrich.com/, or Koch-Light) or Driselase (Sigma-Aldrich) were used in place of pure EPG.

For preparative purposes, AIR of *Rosa* culture was treated with Na_2_CO_3_, then EPG, as above, and the RG-II was purified from the crude digest by gel-permeation chromatography on Bio-Gel P-30 followed by Bio-Gel P-2 (Bio-Rad). The columns were eluted with pyridine/acetic acid/water, 1:1:98, containing 0.5% chlorobutanol. Four independent preparations of *Rosa* RG-II (A–D) were compared.

Red wine was dried and the residue re-dissolved at 1% (w/v) in water. Some samples were then dialysed for 24 h in ‘12-kDa cut-off’ tubing, which removed essentially all the monosaccharide GalA but retained the majority of the (dimeric) RG-II.

### *In-vitro* monomerisation and dimerisation of RG-II

For monomerisation, Arabidopsis RG-II was incubated for 16 h in 0.1 m HCl at 20°C, then de-salted on Bio-Gel P-2 in water. In a study of *in-vitro* dimerisation, the monomer was incubated for 16 h in 0, 0.12 or 1.2 mm H_3_BO_3_, with or without 0.5 mm PbNO_3_. The solutions were buffered at pH 3.0, 5.0 or 7.0 with HEPES, **2**-(*N*-morpholino)ethanesulphonic acid (MES) and acetic acid (50 mm each; Na^+^). Samples were analysed by PAGE without further preparation; the presence of Pb^2+^ and the presence of these buffers did not interfere in the electrophoresis of RG-II.

### Radiolabelling of RG-II

NaB^3^H_4_ (78 MBq; 3.9 GBq μmol^−1^; DuPont, http://www.dupont.com/) in 20 μl 20 mm NaOH was added to 200 μg of RG-II preparation ‘A’ in 100 μl water (neutralised with NaOH) and incubated for 48 h. Xylose (1 mg) was then added and incubated for 5 h to scavenge any remaining NaB^3^H_4_. Next 20 μl of 5% acetic acid was added, and the products were fractionated on Bio-Gel P-10; the void volume (crude [^3^H]RG-II) was collected, repurified on Bio-Gel P-30, monomerised with HCl and desalted on Bio-Gel P-2, as above. The specific activity of the [^3^H]RG-II was estimated (by scintillation-counting and total carbohydrate assay) at 17 MBq μmol^−1^ RG-II monomer.

### Detection of radioactivity

For fluorography, polyacrylamide gels were bathed in glacial acetic acid for 5 min, then in 20% (w/v) 2,5-diphenyloxazole (PPO) in acetic acid for 30 min, rinsed with water for 5 min, dried between cellophane sheets and exposed to pre-flashed film for 1–8 weeks. For quantification of [^3^H]RG-II in gels, the bands were cut out of the dried gel and incubated in 1 ml 2 m TFA at 100°C for 1 h; this hydrolyses the polysaccharide and elutes the radioactive sugars from the gel. Water-miscible scintillation fluid was then added, and ^3^H was assayed in a scintillation counter.

### Sugar analysis

For the analysis of sugar composition, RG-II was hydrolysed in 2 m TFA at 120°C for 1 h and the products were resolved on Merck microcrystalline cellulose TLC plates (http://www.merck.com/) in butanol/acetic acid/water (3:1:1) followed by ethyl acetate/pyridine/water (10:4:3). After staining with aniline hydrogen phthalate (Fry, 2000), the plate was photographed under visible light and 360-nm ultraviolet light.

Additional portions were analysed by HPLC on CarboPac PA1 (Dionex UK, http://www.dionex.com/) eluted at 1 ml min^−1^ with: 0–2 min, 20 mm NaOH; 2–40 min, water; 40–75 min, water → 800 mm NaOH (linear gradient); 75–82 min, 800 mm NaOH; 82–90 min, 20 mm NaOH. A pulsed amperometric detector with a gold electrode was used.

### Tracking the dimerisation of endogenous RG-II domains *in vivo*

*Rosa* cells maintained in B-free medium for at least 8 weeks were re-fed H_3_BO_3_ to 3.3 μm 7 days after subculture. In some cases the cells were in their standard medium and flasks, and normal shaking (aeration) was continued after the addition of H_3_BO_3_. In others, the cells were maintained in carbon-free medium for 4 days before H_3_BO_3_ re-feeding. Alternatively, CCCP or DNP was added from an ethanolic stock solution to give 10 or 200 μm, respectively (accompanied by 0.1% v/v ethanol) at the same time as the H_3_BO_3_; controls received ethanol only. Further 18-ml aliquots of a 7-day culture were dispensed into 60-ml Sterilin beakers; under these conditions, in which the medium has a low surface area:volume ratio, the cultures are partially anaerobic and although remaining viable do not grow. Other 18-ml aliquots were killed by freezing/thawing or by incubation at 100°C for 1 h. In each case, aliquots of culture were taken at intervals after the addition of H_3_BO_3_ and used for the preparation of AIR, treated with Na_2_CO_3_, water and EPG, and the RG-II generated was analysed by PAGE.

### Tracking the possible dimerisation of exogenous free RG-II *in vivo*

For the experiment shown in Figure 6, 150 μl of 4-day-old *Rosa* culture (or cell-free spent medium thereof) was incubated with shaking (aeration) in the presence of about 50 μg of monomerised non-radioactive RG-II (giving about 60 μm) plus 1.2 mm H_3_BO_3_. At intervals, 8-μl samples of medium were removed, frozen and later subjected to PAGE.

To test the fate of lower concentrations of exogenous RG-II in *Rosa* cultures (Figures 7a,b and S2), we fed monomerised [^3^H]RG-II (final concentration 3.9 μm) followed by H_3_BO_3_ (to 1.2 mm) to 150 μl of 4-day-old B-free *Rosa* culture (or cell-free spent medium) and incubated it under standard conditions. At intervals, samples of medium were removed, frozen and subjected to PAGE; the gel was fluorographed, and the radioactive bands were quantified by scintillation counting.

In a separate experiment (Fig. 7c), 8.5 nm [^3^H]RG-II was fed to B-sufficient and B-deficient *Rosa* cultures, with or without 3.3 μm H_3_BO_3_ supplementation. At intervals, samples of medium were assayed for the remaining soluble ^3^H. At 24 h, replicate samples of medium were assayed for total ^3^H and for non-volatile ^3^H remaining after drying and redissolving in the original volume of water (loss of ^3^H on drying indicates any ^3^H_2_O formed).

## Footnotes

* Standard sugar abbreviations are used throughout: Api, d-apiose; Ara, l-arabinose; Dha, 3-deoxy-d-*lyxo*-heptulosaric acid; Fuc, l-fucose; *f*, furanose; d-Gal, d-galactose; l-Gal, l-galactose; GalA, d-galacturonic acid; Glc, d-glucose; GlcA, d-glucuronic acid; Kdo, 2-keto-3-deoxy-d-*manno*-octulosonic acid; MeFuc, 2-*O*-methy-l-fucose; MeXyl, 2-*O*-methyl-d-xylose; *p*, pyranose; Rha, l-rhamnose; Rib, d-ribose; Xyl, d-xylose.

## SUPPORTING INFORMATION

**Figure S1.** Characterisation of purified *Rosa* rhamnogalacturonan-II by HPLC. **Figure S2.** Exogenous [^3^H] rhamnogalacturonan-II fails to dimerise in spent medium of cultured *Rosa* cells.
