# Supplementary material for: Boron bridging of rhamnogalacturonan-II, monitored by gel electrophoresis, occurs during polysaccharide synthesis and secretion but not post-secretion
Source: Plant J. 2013 Dec 9;77(4):534–46. doi: 10.1111/tpj.12403 (PMC4171739; doi:10.1111/tpj.12403)
Supplement: Supplementary file 1 [file tpj0077-0534-SD1.pptx]

## Slide 1
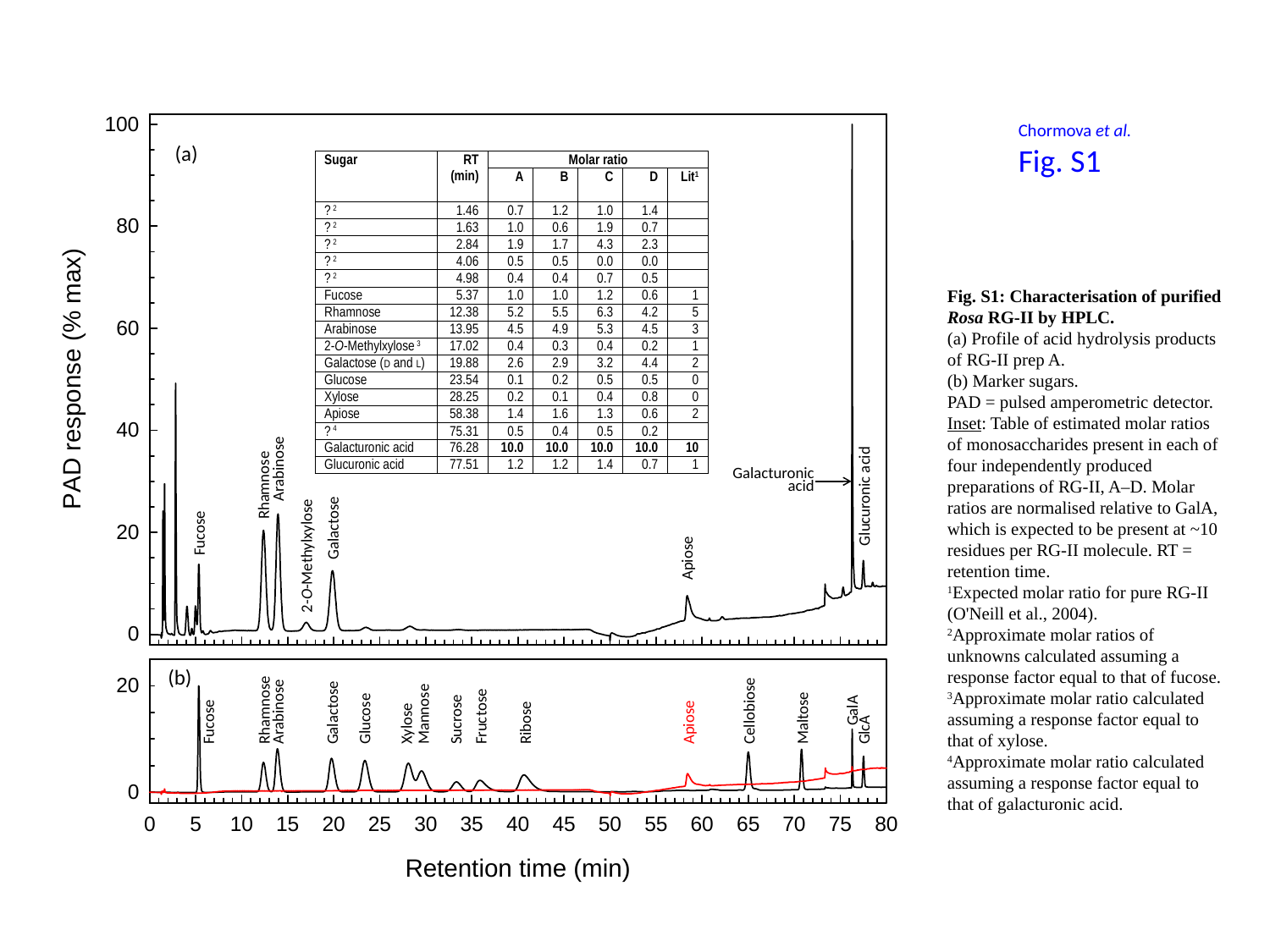

Chormova et al.
Fig. S1
(a)
| Sugar | RT (min) | Molar ratio | | | | |
| --- | --- | --- | --- | --- | --- | --- |
| | | A | B | C | D | Lit1 |
| ? 2 | 1.46 | 0.7 | 1.2 | 1.0 | 1.4 | |
| ? 2 | 1.63 | 1.0 | 0.6 | 1.9 | 0.7 | |
| ? 2 | 2.84 | 1.9 | 1.7 | 4.3 | 2.3 | |
| ? 2 | 4.06 | 0.5 | 0.5 | 0.0 | 0.0 | |
| ? 2 | 4.98 | 0.4 | 0.4 | 0.7 | 0.5 | |
| Fucose | 5.37 | 1.0 | 1.0 | 1.2 | 0.6 | 1 |
| Rhamnose | 12.38 | 5.2 | 5.5 | 6.3 | 4.2 | 5 |
| Arabinose | 13.95 | 4.5 | 4.9 | 5.3 | 4.5 | 3 |
| 2-O-Methylxylose 3 | 17.02 | 0.4 | 0.3 | 0.4 | 0.2 | 1 |
| Galactose (D and L) | 19.88 | 2.6 | 2.9 | 3.2 | 4.4 | 2 |
| Glucose | 23.54 | 0.1 | 0.2 | 0.5 | 0.5 | 0 |
| Xylose | 28.25 | 0.2 | 0.1 | 0.4 | 0.8 | 0 |
| Apiose | 58.38 | 1.4 | 1.6 | 1.3 | 0.6 | 2 |
| ? 4 | 75.31 | 0.5 | 0.4 | 0.5 | 0.2 | |
| Galacturonic acid | 76.28 | 10.0 | 10.0 | 10.0 | 10.0 | 10 |
| Glucuronic acid | 77.51 | 1.2 | 1.2 | 1.4 | 0.7 | 1 |
Fig. S1: Characterisation of purified Rosa RG-II by HPLC.
(a) Profile of acid hydrolysis products of RG-II prep A.
(b) Marker sugars.
PAD = pulsed amperometric detector.
Inset: Table of estimated molar ratios of monosaccharides present in each of four independently produced preparations of RG-II, A–D. Molar ratios are normalised relative to GalA, which is expected to be present at ~10 residues per RG-II molecule. RT = retention time.
1Expected molar ratio for pure RG-II (O'Neill et al., 2004).
2Approximate molar ratios of unknowns calculated assuming a response factor equal to that of fucose.
3Approximate molar ratio calculated assuming a response factor equal to that of xylose.
4Approximate molar ratio calculated assuming a response factor equal to that of galacturonic acid.
Arabinose
Galacturonic
acid
Rhamnose
Glucuronic acid
Galactose
Fucose
2-O-Methylxylose
Apiose
(b)
Rhamnose
GalA
Cellobiose
Arabinose
Galactose
Mannose
Fructose
Maltose
Glucose
Sucrose
Fucose
Apiose
Ribose
Xylose
GlcA

## Slide 2
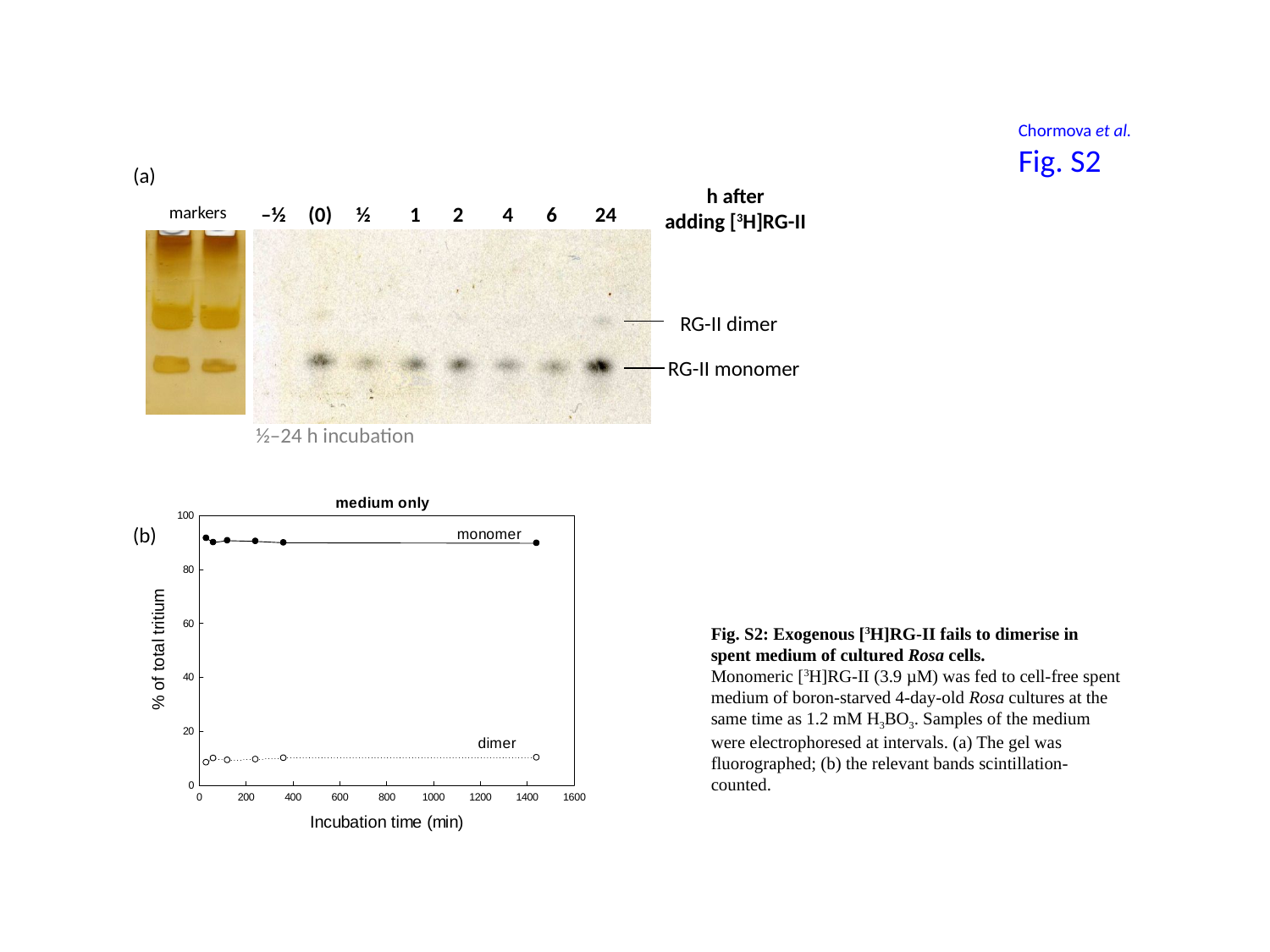

Chormova et al.
Fig. S2
(a)
h after
adding [3H]RG-II
–½
(0)
½
1
2
4
6
24
markers
RG-II dimer
RG-II monomer
½–24 h incubation
(b)
Fig. S2: Exogenous [3H]RG-II fails to dimerise in spent medium of cultured Rosa cells.
Monomeric [3H]RG-II (3.9 µM) was fed to cell-free spent medium of boron-starved 4-day-old Rosa cultures at the same time as 1.2 mM H3BO3. Samples of the medium were electrophoresed at intervals. (a) The gel was fluorographed; (b) the relevant bands scintillation-counted.
