# Supplementary material for: Boron bridging of rhamnogalacturonan-II, monitored by gel electrophoresis, occurs during polysaccharide synthesis and secretion but not post-secretion
Source: Plant J. 2013 Dec 9;77(4):534–46. doi: 10.1111/tpj.12403 (PMC4171739; doi:10.1111/tpj.12403)
Supplement: Supplementary file 2 [file tpj0077-0534-SD2.docx]

**Titles + legends for the supporting information**

**Fig. S1: Characterisation of purified *Rosa* RG-II by HPLC.**

(a) Profile of acid hydrolysis products of RG-II prep A.

(b) Marker sugars.

PAD = pulsed amperometric detector.

Inset: Table of estimated molar ratios of monosaccharides present in each of four independently produced preparations of RG-II, A–D. Molar ratios are normalised relative to GalA, which is expected to be present at ~10 residues per RG-II molecule. RT = retention time.

^1^Expected molar ratio for pure RG-II (O’Neill et al., 2004).

^2^Approximate molar ratios of unknowns calculated assuming a response factor equal to that of fucose.

^3^Approximate molar ratio calculated assuming a response factor equal to that of xylose.

^4^Approximate molar ratio calculated assuming a response factor equal to that of galacturonic acid.

**Fig. S2: Exogenous [^3^H]RG-II fails to dimerise in spent medium of cultured *Rosa* cells.**

Monomeric [^3^H]RG-II (3.9 µM) was fed to cell-free spent medium of boron-starved 4-day-old *Rosa* cultures at the same time as 1.2 mM H_3_BO_3_. Samples of the medium were electrophoresed at intervals. (a) The gel was fluorographed; (b) the relevant bands scintillation-counted.
